# Supplementary material for: A Machine Learning Framework Based on Extreme Gradient Boosting to Predict the Occurrence and Development of Infectious Diseases in Laying Hen Farms, Taking H9N2 as an Example
Source: Animals (Basel). 2023 Apr 27;13(9):1494. doi: 10.3390/ani13091494 (PMC10177545; doi:10.3390/ani13091494)
Supplement: Supplementary file 1 [file animals-13-01494-s001.zip › Codes S1.pdf]

Codes S1: Python codes

Take "laying rate and mortality of Day 0 as input, H9N2 status+0 as output" as an example, the Python codes are:

```
# Import packages
import pandas as pd
import numpy as np
import os
from matplotlib import pyplot as plt

from sklearn.model_selection import train_test_split
from sklearn.ensemble import RandomForestRegressor, RandomForestClassifier
from sklearn.neural_network import MLPRegressor, MLPClassifier
from sklearn.linear_model import LinearRegression, LogisticRegression
from sklearn.svm import SVR
import xgboost as xgb

from scipy.stats import pearsonr
from sklearn.metrics import accuracy_score, explained_variance_score,
mean_squared_error,recall_score
from sklearn.model_selection import GridSearchCV
import warnings
warnings.filterwarnings("ignore")

root_folder = os.getcwd()
raw_data = pd.read_csv(r'-4~0.csv',encoding="ISO-8859-1")

X_raw = raw_data[['lay_0','death_0']]
X_raw
```

```
y_raw= raw_data['illness0']

X = X_raw[0:72]
X_test = X_raw[73:112]
y = y_raw[0:72]
y_test = y_raw[73:112]

param_test1 = {
 'n_estimators':list(range(0,100,1)),
#   'max_depth':list(range(1,15,1)),
#   'min_child_weight':list(range(1,15,1))
}
gsearch1 = GridSearchCV(estimator = xgb.XGBClassifier( learning_rate =0.1,
n_estimators=13, max_depth=4,
 min_child_weight=1, gamma=0, subsample=0.8, colsample_bytree=0.8,
 objective= 'binary:logistic', nthread=4, scale_pos_weight=1, seed=27),
 param_grid = param_test1, scoring='accuracy', cv=5)
gsearch1=gsearch1.fit(X,y)
gsearch1.best_params_, gsearch1.best_score_

param_test2 = {
#   'n_estimators':list(range(0,100,1)),
 'max_depth':list(range(1,15,1)),
 'min_child_weight':list(range(1,15,1))
}
gsearch1 = GridSearchCV(estimator = xgb.XGBClassifier( learning_rate =0.1,
n_estimators=72, max_depth=4,
 min_child_weight=1, gamma=0, subsample=0.8, colsample_bytree=0.8,
 objective= 'binary:logistic', nthread=4, scale_pos_weight=1, seed=27),
 param_grid = param_test2, scoring='accuracy', cv=5)
```

```
gsearch1=gsearch1.fit(X,y)
gsearch1.best_params_, gsearch1.best_score_

param_test3 = {
#    'n_estimators':list(range(0,100,1)),
#    'max_depth':list(range(1,15,1)),
#    'min_child_weight':list(range(1,15,1)),
        'gamma':[i/10.0 for i in range(0,10)]

}
gsearch1 = GridSearchCV(estimator = xgb.XGBClassifier( learning_rate =0.1, n_estimators=72, max_depth=3,
  min_child_weight=1, gamma=0, subsample=0.8, colsample_bytree=0.8,
  objective= 'binary:logistic', nthread=4, scale_pos_weight=1, seed=27),
  param_grid = param_test3, scoring='accuracy', cv=5)
gsearch1=gsearch1.fit(X,y)
gsearch1.best_params_, gsearch1.best_score_

param_test4 = {
#    'n_estimators':list(range(0,100,1)),
#    'max_depth':list(range(1,15,1)),
#    'min_child_weight':list(range(1,15,1)),
#          'gamma':[i/10.0 for i in range(0,10)]
          'subsample':[i/100.0 for i in range(70,90,5)],
  'colsample_bytree':[i/100.0 for i in range(70,90,5)]
}
gsearch1 = GridSearchCV(estimator = xgb.XGBClassifier( learning_rate =0.1, n_estimators=72, max_depth=3,
  min_child_weight=1, gamma=0, subsample=0.8, colsample_bytree=0.8,
  objective= 'binary:logistic', nthread=4, scale_pos_weight=1, seed=27),
```

```
  param_grid = param_test4, scoring='accuracy', cv=5)
gsearch1=gsearch1.fit(X,y)
gsearch1.best_params_, gsearch1.best_score_

param_test5 = {
#    'n_estimators':list(range(0,100,1)),
#    'max_depth':list(range(1,15,1)),
#    'min_child_weight':list(range(1,15,1)),
#         'gamma':[i/10.0 for i in range(0,10)],
#           'subsample':[i/100.0 for i in range(70,90,5)],
#    'colsample_bytree':[i/100.0 for i in range(70,90,5)],
         'learning_rate':[0.001,0.01, 0.02, 0.1, 0.2]
}
gsearch1 = GridSearchCV(estimator = xgb.XGBClassifier( learning_rate =0.1, n_estimators=72, max_depth=3,
  min_child_weight=1, gamma=0.0, subsample=0.75, colsample_bytree=0.7,
  objective= 'binary:logistic', nthread=4, scale_pos_weight=1, seed=27),
  param_grid = param_test5, scoring='accuracy', cv=5)
gsearch1=gsearch1.fit(X,y)
gsearch1.best_params_, gsearch1.best_score_

model = xgb.XGBClassifier(binary = 'logistic',learning_rate =0.1, n_estimators=72, max_depth=3,
  min_child_weight=1, gamma=0, subsample=0.75, colsample_bytree=0.7,objective= 'binary:logistic')

model.fit(X,y)

y_pred = model.predict(X_test)
```

```
true = np.sum(y_pred == y_test )
print('The correct result is：', true)
print('The wrong result is：', y_test.shape[0]-true)
from sklearn.metrics import
accuracy_score,precision_score,recall_score,f1_score,cohen_kappa_score
print('The accuracy of the predicted data is ：
{:.4}%'.format(accuracy_score(y_test,y_pred)*100))
print('The precision of the predicted data is：{:.4}%'.format(
        precision_score(y_test,y_pred)*100))
print('The recall rate of the predicted data is：{:.4}%'.format(
        recall_score(y_test,y_pred)*100))
# print("The F1 value of the predicted data is：", f1score_train)
print('The F1 value of the predicted data is：',
        f1_score(y_test,y_pred))
print('The Cohen’s Kappa factor of the predicted data is：',
        cohen_kappa_score(y_test,y_pred))

from xgboost import plot_importance

plot_importance(model)

from sklearn.metrics import roc_curve
y_pred_proba = model.predict_proba(X_test)
fpr,tpr,thres = roc_curve(y_test,y_pred_proba[:,1])
import matplotlib.pyplot as plt
plt.plot(fpr,tpr)
plt.show()

from sklearn.metrics import precision_recall_curve
from sklearn import metrics
```

```
y_pred_prob=model.predict_proba(X_test)[:,1]
fpr, tpr, thresholds = metrics.roc_curve(y_test,y_pred_prob, pos_label=1)
roc_auc = metrics.auc(fpr, tpr)
plt.figure(figsize=(8,6))
plt.plot([0, 1], [0, 1], color='navy', lw=2, linestyle='--')
plt.plot(fpr, tpr, 'r',label='AUC = %0.2f'% roc_auc)
plt.legend(loc='lower right')
plt.xlim([0, 1.1])
plt.ylim([0, 1.1])
plt.xlabel('False Positive Rate')
plt.ylabel('True Positive Rate')
plt.title('Receiver operating characteristic example')
plt.show()

fpr
tpr
```
