# Supplementary material for: A Machine Learning Framework Based on Extreme Gradient Boosting to Predict the Occurrence and Development of Infectious Diseases in Laying Hen Farms, Taking H9N2 as an Example
Source: Animals (Basel). 2023 Apr 27;13(9):1494. doi: 10.3390/ani13091494 (PMC10177545; doi:10.3390/ani13091494)
Supplement: Supplementary file 1 [file animals-13-01494-s001.zip › Datasets S1.pdf]

Datasets S1: Part of the collated datasets using in this study

	laying rate_-4	laying rate_-3	laying rate_-2	laying rate_-1	laying rate_0	mortality_-4	mortality_-3	mortality_-2	mortality_-1	mortality_0	H9N2 status 0	H9N2 status 1	H9N2 status 2
1	97.1	97.1	96.3	95.5	95.5	0.02	0.03	0.03	0.03	0.02	0	0	0
2	97.1	96.3	95.5	95.5	95.6	0.03	0.03	0.03	0.02	0.04	0	0	0
3	96.3	95.5	95.5	95.6	95.6	0.03	0.03	0.02	0.04	0.02	0	0	0
4	95.5	95.5	95.6	95.6	96	0.03	0.02	0.04	0.02	0.04	0	0	0
5	95.5	95.6	95.6	96	96	0.02	0.04	0.02	0.04	0.05	0	0	0
6	95.6	95.6	96	96	94	0.04	0.02	0.04	0.05	0.05	0	0	0
7	95.6	96	96	94	92.4	0.02	0.04	0.05	0.05	0.06	0	0	0
8	96	96	94	92.4	91	0.04	0.05	0.05	0.06	0.08	0	0	1
9	96	94	92.4	91	90.5	0.05	0.05	0.06	0.08	0.1	0	1	1
10	94	92.4	91	90.5	89.2	0.05	0.06	0.08	0.1	0.05	1	1	1
11	92.4	91	90.5	89.2	87.6	0.06	0.08	0.1	0.05	0.05	1	1	1
12	89.2	87.6	87.4	88.3	89	0.05	0.05	0.05	0.05	0.03	1	1	1
13	87.6	87.4	88.3	89	89.7	0.05	0.05	0.05	0.03	0.05	1	1	1
14	87.4	88.3	89	89.7	90.3	0.05	0.05	0.03	0.05	0.03	1	1	1
15	89.7	90.3	91.3	92.1	92.3	0.05	0.03	0.03	0.03	0.03	1	1	1
16	90.3	91.3	92.1	92.3	92.8	0.03	0.03	0.03	0.03	0.03	1	1	0
17	91.3	92.1	92.3	92.8	93.2	0.03	0.03	0.03	0.03	0.02	1	0	0
18	92.1	92.3	92.8	93.2	92.4	0.03	0.03	0.03	0.02	0.04	0	0	0
19	94.34	94.3	94.26	94.29	94.15	0.03	0.04	0.03	0.03	0.03	0	0	0
20	94.3	94.26	94.29	94.15	94.15	0.04	0.03	0.03	0.03	0.04	0	0	0

For the result to be practical for field scientists and researchers without bioinformatic/machine learning skills, we take "part of the collated datasets using for prediction model development in this study" as an example to help readers easily understand how to prepare the datasets directly used to input the model.
