# Supplementary material for: A Machine Learning Framework Based on Extreme Gradient Boosting to Predict the Occurrence and Development of Infectious Diseases in Laying Hen Farms, Taking H9N2 as an Example
Source: Animals (Basel). 2023 Apr 27;13(9):1494. doi: 10.3390/ani13091494 (PMC10177545; doi:10.3390/ani13091494)
Supplement: Supplementary file 1 [file animals-13-01494-s001.zip › Figure S1.pdf]

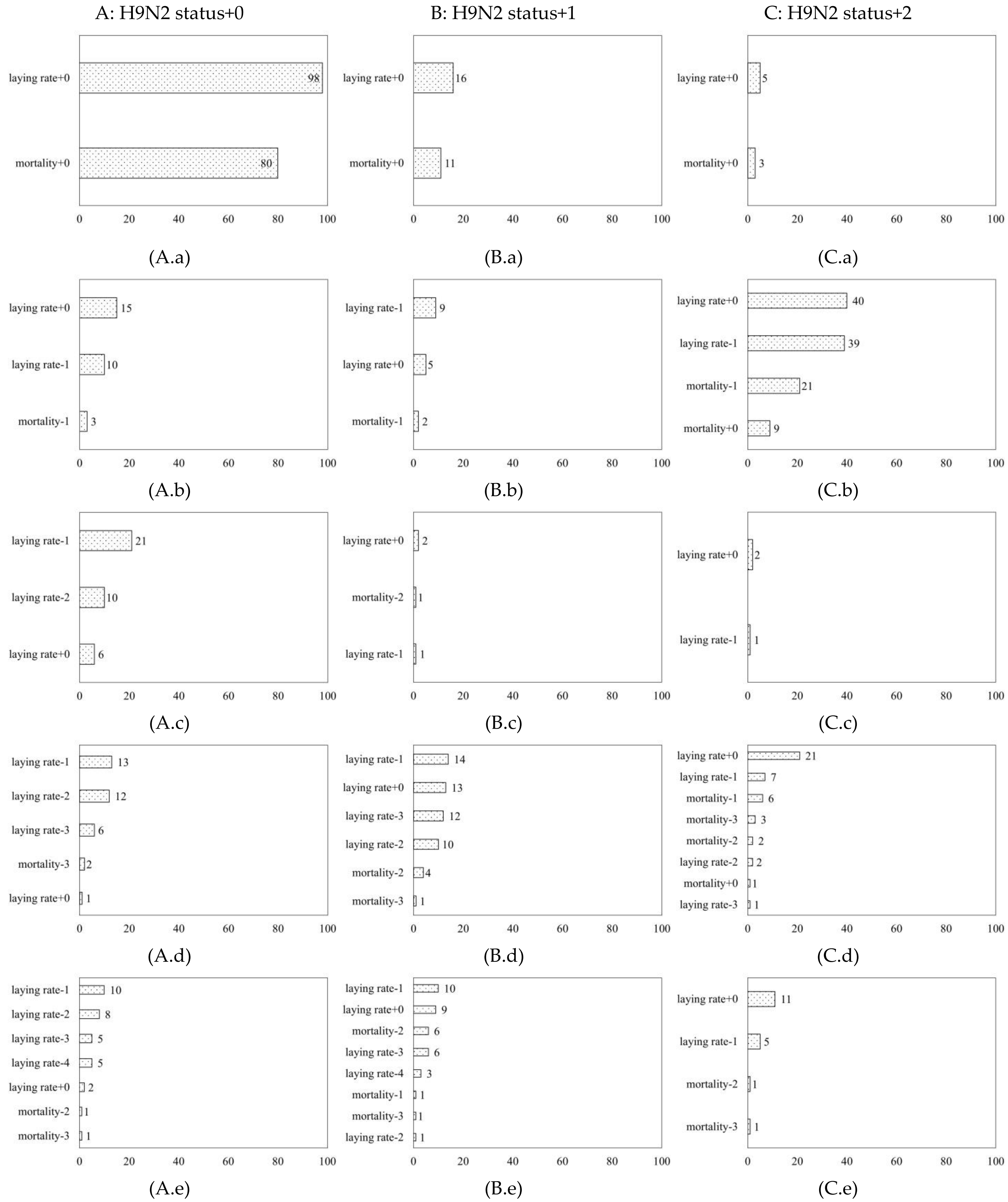

Figure S1. Feature importance for H9N2 status+0 (A), +1 (B), +2 (C) predictions in laying hen houses from the XGBoost classification models with time frames of day+0 (a), day-1 (b), day-2 (c), day-3 (d), and day-4 (e). +2 = future 2nd day, +1 = future 1st day, +0 = today, -1 = past 1 day, -2 = past 2 days, -3 = past 3 days, and -4 = past 4 days.
